# Food webs can deliver win–win strategies for tropical agroforestry and biodiversity conservation

**DOI:** 10.1002/eap.70093

**Published:** 2025-09-12

**Authors:** Crinan Jarrett, Luke L. Powell, Tabe Tiku Regine Claire, Cyril Kowo, Diogo F. Ferreira, Alma L. S. Quiñones, Andreanna J. Welch, Daniel T. Haydon, Jason Matthiopoulos

**Affiliations:** ^1^ Bird Migration Unit Swiss Ornithological Institute Luzern Switzerland; ^2^ School of Biodiversity, One Health and Veterinary Medicine University of Glasgow Glasgow UK; ^3^ Biodiversity Initiative Houghton Michigan USA; ^4^ CIBIO, Centro de Investigação em Biodiversidade e Recursos Genéticos, InBIO Laboratório Associado, Universidade do Porto Porto Portugal; ^5^ BIOPOLIS Program in Genomics, Biodiversity and Land Planning, CIBIO Porto Portugal; ^6^ Department of Microbiology and Parasitology University of Buea Buea Southwest Region Cameroon; ^7^ Department of Biosciences Durham University Durham UK

**Keywords:** arthropods, birds, cocoa, community modeling, diet metabarcoding, ecosystem management, pests, population dynamics

## Abstract

High biodiversity and agricultural productivity are commonly regarded as mutually exclusive. However, functionally diverse communities may provide valuable services to agroecosystems and therefore offer the possibility of win–win strategies. We developed a dynamic mechanistic community model of the bird–arthropod food web associated with African cocoa agroforestry, structurally informed by metabarcoding data on bird diets, and fitted to trapping data on species abundances. Our novel framework models rates of change and uses space‐for‐time substitution, thus providing insights into community dynamics without the need for long time‐series data. We used our fitted model to predict equilibrium community composition under varying intensities of shade management and pesticide use. Our results indicate that low‐intensity farming favors forest bird species, with at least two times the biomass of this bird group compared to any other at high shade cover. Low‐intensity farms also favored potential pollinator abundance, while biomass of the main pest species of cocoa, brown capsid, was 25% lower at high shade than at low shade. Our model quantified the net effect of each taxon on the other taxa in the food web: most bird to arthropod interactions were negative, indicating important pest control services provided by birds. Furthermore, our simulations of pesticide application revealed that the long‐term effect of pesticide use on biomass of taxa varied according to shade cover. Importantly, pesticide application resulted in the decline of non‐pest taxa through trophic cascades: forest birds were the taxa that declined the fastest, and this trend was exacerbated in low shade farms. To achieve a decline of less than 50% in non‐pest taxa, pesticide application could only reach 10% in the sunniest farms and 20% in shady farms, which results in a maximal reduction of 19% in pest biomass. By looking at the efficacy of agricultural management through the lens of community interactions, our holistic, quantitative approach demonstrates that low‐intensity agriculture may provide a win–win for biodiversity and ecosystem services.

## INTRODUCTION

Low‐intensity small‐holder agriculture is widely practiced in the tropics, where some of the world's economically poorest but biodiversity‐richest regions are located (Tscharntke et al., 2012). In these areas, farmers commonly live on <1€/day, surrounded by some of the highest levels of wildlife diversity on Earth (Tscharntke et al., 2012). With limited resources to manage their land, farmers in tropical regions often rely heavily on hand‐held tools and ecosystem services provided by wildlife (Tscharntke et al., 2011). Therefore, low‐intensity agriculture offers opportunities for both agricultural productivity and biodiversity conservation (Clough et al., 2011; Tscharntke et al., 2011). However, it remains unclear how to manage these systems to achieve optimal combinations of the ostensibly competing aims of sustainable agricultural yields and biodiversity conservation.

The growing demand for food is pressurizing policymakers into encouraging agricultural expansion and intensification (Ordway et al., 2017). Consequently, effectively managed wildlife‐friendly agriculture is more important now than ever (Tscharntke et al., 2012). Intensification of agriculture commonly results in the replacement of floristically diverse agroecosystems with monocultures, often treated with high levels of chemical inputs (Clough et al., 2009; Tscharntke et al., 2011). This intensification results in community changes, loss of biodiversity, and abundance declines (Cassano et al., 2009; De Beenhouwer et al., 2013; Ferreira, Darling, et al., 2023; Jarrett et al., 2021). The impoverishment of wildlife communities could also lead to unexpected loss of agricultural productivity, for instance, due to reductions in animal groups that provide ecosystem services, such as pollination and pest control (Maas et al., 2013, 2016).

Understanding the trade‐offs and synergies between biodiversity and productivity is complex and has rarely been approached from a community‐wide perspective (but see Karp & Daily, 2014; Kean et al., 2003; Kross et al., 2011). Animal communities in low‐intensity agroecosystems are diverse and contain species that influence productivity and biodiversity conservation outputs in different ways (Bagny Beilhe et al., 2018; Maas et al., 2013; Toledo‐Hernández et al., 2021). Species that affect productivity either directly (e.g., pollinators) or indirectly (e.g., predators of pollinators or pests) are broadly considered to provide ecosystem services or disservices (but see Pascual et al., 2017, for discussion of updated terminology). In our agroecological context, we consider disservices to be processes caused by wildlife that have a negative impact on agricultural production, such as losses in harvests due to insect pests. Different community compositions of fauna therefore result in different outcomes for biodiversity conservation, ecosystem services, and disservices in agroecosystems. Ideally, systems would be managed to optimize a combination of desirable outcomes, for instance, high abundance of species of conservation value and ecosystem service providers, and low abundance of disservice providers. However, because each species can respond differently to habitat management, creating a potential cascade of direct and indirect effects of management on the whole food web, responses of communities to agricultural management are hard to predict.

Previous research into the effect of agricultural management on biodiversity or productivity has mostly focused on establishing correlations between the observed abundance of different groups and management covariates (Blaser et al., 2018; Clough et al., 2011). The limitation with these correlative analyses is that they commonly assume that species respond to management independently from each other. In other words, a correlation is established between the observed density of a species and management covariates, without accounting for the fact that this species' density depends on a complex network of interactions. Thus, densities of other species in the community present may be essential in determining community states (Gotelli & Ellison, 2006; Janssen & van Rijn, 2021; Kawatsu et al., 2021; Tylianakis et al., 2007). To accurately predict the effects of management on wildlife populations, and consequently on biodiversity conservation, ecosystem services, and disservices, we need a framework that can incorporate both direct responses of species to management and interactions between species in the food web.

Mechanistic community models could provide this framework. However, the common challenge with such models is that they involve many parameters and are consequently hard to fit to short‐term field study data (Burt et al., 2018; Curtsdotter et al., 2019; Kawatsu et al., 2021). Community models are typically believed to require long time series of data on ecological communities in order to distinguish the noise (demographic and environmental stochasticity) from the parameters governing the composition of communities (Ellner et al., 2002; Yodzis, 1998). However, dynamical models (both difference and differential equation models) focus on rates of change, not absolute abundances. Information on rates of change does not necessarily require long time series: pairs of successive observations may be sufficient (Ives et al., 2003). Here, we sought to investigate fitting complex mechanistic community models to short time series by exploiting a space‐for‐time substitution; we considered paired observations from a range of sites with shared parameters so that community composition at time *t* depended only on community composition at time *t* − 1. This experimental design is common in ecological studies, making our modeling framework applicable to datasets derived from typical field projects of typical 2‐ to 3‐year duration and particularly suitable for pooling multiple data fragments into a common inferential platform.

We modeled complex community dynamics using a set of simultaneous difference equations, based on concepts from traditional community ecology, but formulated as a series of coupled GLMs, enabling us to directly fit them to field data using Bayesian methods. Our model incorporates data on species' abundances and considers rates of change in the density of each species as a function of environmental covariates (including management). We informed trophic links between species through a dataset of >300 diet samples from insectivorous birds, processed using diet metabarcoding. This is, to our knowledge, the largest high‐resolution dataset on Afrotropical bird diets and provides novel and robust data on trophic connections between birds and arthropods in our system. Our models capture full‐system dynamics, incorporating both the direct and indirect effects of management and species on each other. Importantly, processes in agricultural habitats are influenced not only by local management but also by landscape‐scale effects. The diversity of species in an agroecosystem and the functions these species carry out, such as pollination and pest control, are dependent on the dynamics of source populations and the potential connectivity between sites (Edwards et al., 2021; Ferreira et al., 2024; Jarrett et al., 2021). This aspect, while important in agroecology, is thus far not incorporated into our food web framework; however, we discuss below how such processes could be included.

Agroforestry, where crops are grown under a canopy of shade trees, provides an ideal model system to investigate community dynamics and resulting ecosystem services and disservice outcomes. Such systems are a common form of agricultural production in tropical regions, can be highly diverse, and contain many service‐ and disservice‐providing species. Within agroforestry, the insectivore–insect food web is central and reflects important synergies and trade‐offs between the conservation of vulnerable taxa and the provisioning of ecosystem services (e.g., crop pollination) and disservices (e.g., crop pests); insectivores, especially avian insectivores, are common in agroforestry (Jarrett et al., 2021, 2022) and can play an important role in pest control (Ferreira, Jarrett, et al., 2023; Maas et al., 2016). Additionally, insectivores are vulnerable to habitat degradation and consequently should be a priority for conservation in these landscapes (Bregman et al., 2014; Jarrett et al., 2021; Powell et al., 2015). Arthropods are a widespread group in agroforestry and can be beneficial service providers (pollination, pest control); however, they can also be extremely damaging to crops, causing ecosystem disservices that can amount to crop losses of up to 40% (Akesse‐Ransford et al., 2021; Bisseleua et al., 2013; Wessel & Quist‐Wessel, 2015).

In this article, we aimed to introduce our modeling framework in general terms, to allow potential applications to different systems, and then to demonstrate its uses when applied to a complex agroecological system, African cocoa agroforestry. Applying our model to cocoa agroforestry data, we aimed to: (a) investigate the effects of management on long‐term community composition (states) and assess the consequences for biodiversity conservation and ecosystem (dis‐)services, (b) estimate and examine the interactions driving food web dynamics and their relative contribution toward community states, and (c) predict changes in community composition under different pesticide application scenarios.

## METHODS

### Modeling framework

#### Conceptual background

**FIGURE 1:**
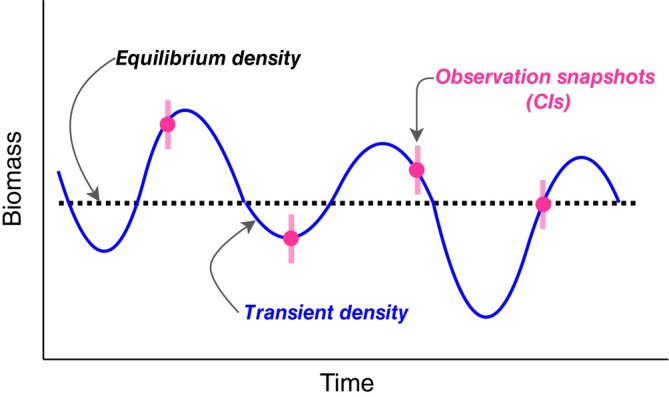
Rationale of our community model: we assume that the biomass of each species fluctuates stochastically (solid blue line) around an equilibrium (dashed black line), which is determined by species' intrinsic growth rates (a function of environmental covariates) and inter‐ and intra‐specific interactions. When we observe species' abundances, we see a snapshot of the transient densities (solid blue line), but observation is inevitably made with error (pink points and strips).

We assumed that communities of species fluctuate around a given (potentially unobserved) equilibrium state (Figure 1). We use the term “species” throughout to describe taxonomic groups, which may be species but could also be functional groups, families, etc. We define community state as the vector of the abundances of all species at a given point in time $N_{t}=\left\{N_{t1},\ldots ,N_{\mathrm{ti}},\ldots ,N_{\mathrm{tI}}\;\right\}$, where *I* is the total number of species, including those that happen to have a zero density in any particular system. While stable equilibria cannot be assumed to be observed in the data (Figure 1), they are latent states toward which a system will tend, so they are the objective of our estimation.

Community states are the result of species' growth, environmental conditions, and interactions between species. Observations of the state of the community were made at discrete timepoints and captured a state of transience around the equilibrium (Figure 1). Given sufficient observations of different transient states, we should be able to differentiate the noise (demographic and environmental stochasticity) from the parameters governing the equilibrium states. Our model was designed to fit a set of paired timepoints (time *t* and *t* + 1), and these pairs could come from different locations characterized by different environmental and management regimes, but were assumed to be governed by shared parameters (albeit some modified covariates). Therefore, species' intrinsic growth rates, trophic interactions, and responses to the environment were assumed to be shared across sites, while species' densities and environmental conditions could vary between sites.

#### General model structure

Our model was based on a discrete‐time Lotka–Volterra community model with *I* potentially interacting species. We modeled biomass of each species (continuous response variable) with a Gamma distribution where variance was equal to the mean (Equation 1), to approximate a Poisson distribution commonly used to model species abundances, but adapted to biomass measurements. The mean was written as a function of biomass at the previous time step and the exponential of a linear predictor $L_{\mathrm{ti}}$, representing per‐capita rate of change for each species at a given time.

(1) $$N_{\left(t+1i\right)}\sim \text{Gamma}\left({\mu_{\mathrm{ti}}}^{2}/\mu_{\mathrm{ti}},1\right)$$

(2) $$\mu_{\mathrm{ti}}=N_{\mathrm{ti}}\exp\left(L_{\mathrm{ti}}\right)$$

The log of the per‐capita rate of change $L_{\mathrm{ti}}$ was written as a linear predictor, with an intercept $b_{i}$ interpreted as the log of the species‐specific intrinsic growth rate (i.e., rate of change in the absence of all other species, whether predators or prey), and the sum of the effects of all interactions between species.

(3) $$L_{\mathrm{ti}}=b_{i}+\left(\sum_{j=1}^{I}a_{\mathrm{ij}}N_{\mathrm{tj}}\right)$$

The coefficients $a_{\mathrm{ij}}$ quantify the effect of the abundance of species *j* on the per‐capita population growth rate of species *i*. Intra‐specific effects (e.g., density dependence) were captured by $a_{\mathrm{ii}}$. We assumed a Holling Type I functional response between predators and prey to simplify model parametrization, but non‐linear functional responses could in theory be used here either approximately, via quadratic terms, or exactly by incorporating type II or III functional responses (at the cost of doubling or trebling the number of model parameters).

In these coupled equations, the equilibrium state was given when $\exp\left(L_{\mathrm{ti}}\right)=1$ for all *i*, and therefore $b_{i}+\sum_{j=1}^{\mathrm{JI}}a_{\mathrm{ij}}N_{\mathrm{tj}}=0$ for all *i*. The equilibrium state can be found by multiplying the inverse of the A matrix (containing the $a_{\mathrm{ij}}$ elements) by the vector of intrinsic growth rates b (containing the $b_{i}$ elements).

(4) $$N^{*}=-A^{-1}b$$

The elements ($-{a_{\mathrm{ij}}}^{-1}$) of the inverse of the **A** matrix $\left(A^{-1}\right)$ capture the overall effect of each taxon on each other, defined as the change in equilibrium biomass of one taxon as a function of sustained increase in the biomass of another (Yodzis, 1988). These parameters therefore represent the combined influence of all direct and indirect effects of changes in biomass of species *j* or species *i*.

#### Environmental covariates

The log of the intrinsic growth rate ($b_{i}$) of species *i* could be influenced by environmental covariates (i.e., independent variables). This linear model can incorporate potential effects of habitat characteristics, climate, or landscape on species' demographic rates.

(5) $$b_{i}=\sum_{q=0}^{Q}\nu_{\mathrm{iq}}X_{q}$$

The linear predictor comprised Q covariates, $X_{q}$, affecting growth rate, and their respective regression coefficients $\nu_{\mathrm{iq}}$, where *q* refers to the *q*th covariate (the intercept $\nu_{i0}$ was included by setting $X_{0}=1$). Including the effect of environmental covariates on $b_{i}$ resulted in a **b** vector whose elements were functions of these covariates. Consequently, equilibrium densities as calculated by Equation (4) changed log‐linearly with environmental covariates.

Before fitting the community model to field data, we tested it on simulated data to assess the accuracy and precision of parameter posteriors (Appendix S1: Section S1) compared to known underlying parameter values.

### Application to cocoa agroforestry

#### Field data collection

**FIGURE 2:**
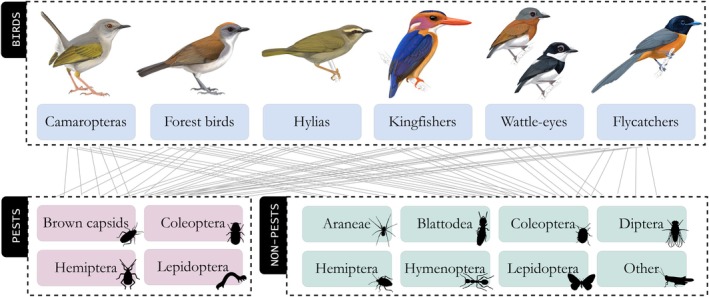
Structure of cocoa farm bird–arthropod food web: two trophic levels representing insectivorous birds (predators) and arthropods (prey). Bird illustrations by Faansie Peacock. Arthropod illustrations were obtained from phylopic.org as follows: Coleoptera pest (credit: Kristina Gagalova) and Lepidoptera non‐pest (credit: Fritz Geller‐Grimm, vectorized by T. Michael Keesey) under CC BY‐SA 3.0 licenses (https://creativecommons.org/licenses/by‐sa/3.0/); Blattodea (credit: Melissa Broussard), Coleoptera non‐pest (credit: Gareth Monger), and Hemiptera non‐pest (credit: Dave Angelini) under CC BY 3.0 licenses (https://creativecommons.org/licenses/by/3.0/); brown capsid, Hemiptera pest, Lepidoptera pest, Araneae, Diptera, Hymenoptera, and Orthoptera under CC0 1.0 licenses (public domain).

We applied our model to field data collected from 28 Cameroonian cocoa farms during January–February and August–September 2019–2020. The farms were on a gradient of shade cover ranging from 19% to 99% and were spread across five landscapes (Appendix S1: Section S2). These landscapes ranged from forest‐dominated areas bordering the Dja Forest Reserve to densely developed landscapes near Yaoundé city (Appendix S1: Section S2). Each farm was visited two to four times, and on each occasion, we surveyed birds and arthropods and collected bird fecal samples for diet analysis. We aggregated species into groups to reduce the number of model parameters. Based on our field data, we determined the main groups (henceforth “taxa”) that formed the bird–arthropod food web in these cocoa farms (Figure 2). For birds, 60% of all insectivorous individuals captured belonged to one of five genera or belonged to a guild of forest specialists (Jarrett et al., 2021), so we clustered our food web accordingly (Appendix S1: Section S3). The genera *Camaroptera*, *Hylia* (*H. prasina*, green hylia), *Platysteira* (wattle‐eyes), and *Terpsiphone* (flycatchers) are small passerine birds, some species of which are sensitive to habitat degradation (Jarrett et al., 2021; Appendix S1: Section S3). *Ispidina* is a genus of small insectivorous kingfishers, considered habitat generalists (Jarrett et al., 2021; Naidoo, 2004). These groups of insectivorous birds likely provide ecosystem services by consuming pest arthropods. We classified arthropods as either “pests” or “non‐pests” and then grouped them by order (Figure 2), except for brown capsid (*Sahlbergella singularis*), the primary pest of cocoa in Africa (Bagny Beilhe et al., 2018), which was included at the species level. Aside from pests, which we consider disservice providers in cocoa agroforestry, several arthropod groups provide ecosystem services such as Diptera (pollinators) and Araneae (natural enemies; Jarrett et al., 2023; Toledo‐Hernández et al., 2021).

##### Birds

The bird dataset used for the model was that of Jarrett et al. (2022). The dataset consisted of bird mist‐net captures and simultaneously collected acoustic recordings from Cameroonian cocoa farms from January to February and August to September 2019–2020 (a period of 19 months). The mist‐netting dataset contained 2011 captures from 118 species, and the acoustic dataset included detections of 148 species. These data were collected across 84 days, with each farm surveyed for one day each visit. All birds caught were identified to species level and weighed to allow conversion to biomass.

##### Arthropods

The arthropod dataset used for the model was identical to that of Jarrett et al. (2023). It included count data for arthropods collected simultaneously with bird data over 84 days, with each farm surveyed once per visit. We used three common survey methods to survey arthropods: sweep‐netting, malaise traps, and visual surveys. Arthropods were identified to order level, except for brown capsid (*Sahlbergella singularis*), the primary pest of cocoa in Africa, which was identified to species level. Aside from brown capsid, we considered three other potential pest groups: Coleoptera, Hemiptera, and Lepidoptera, and the distinction between pests and non‐pests in these orders was made in the field based on empirical observations of individuals damaging cocoa crops (Jarrett et al., 2023).

##### Trophic links

We analyzed fecal samples of 319 insectivorous birds (*n* = 74 camaroptera, *n* = 56 flycatcher, *n* = 28 wattle‐eye, *n* = 65 kingfisher, *n* = 29 hylia, *n* = 67 forest birds) using diet metabarcoding methods to inform trophic links in the food web model (Appendix S1: Section S4). Samples contained on average 3.9 prey taxa identified to order level (SD = 2.7; Appendix S1: Section S4).

##### Covariate (independent variable) data

The 28 cocoa farms sampled were distributed across a gradient of shade cover, ranging from 19% to 99%. Our method for quantifying shade cover (in short, as percentage of the sky obscured by shade trees above the cocoa) is described in Jarrett et al. (2021).

#### Conversion to biomass data

Our modeling framework takes biomass data of species as input, and thus, we needed to convert bird and arthropod counts to biomass. Additionally, we needed to account for imperfect detection in field data. To achieve this, we used the observation models in Jarrett et al. (2022, 2023) for birds and arthropods, respectively (for details, see Appendix S1: Section S5). The bird model combined mark–recapture data from mist‐net captures with detections from acoustic recorders into a joint likelihood to estimate population size at each site. In this model, site is considered to be the 1.5‐ha area covered by the sampling radius of the mist‐nets, and thus, the estimated bird population represents the number of birds whose home ranges overlap with this area (Jarrett et al., 2022). We then extrapolated bird population size to 1 km^2^. In addition, we selected from the output of this observation model only the taxa considered in the food web (camaropteras, hylias, kingfishers, wattle‐eyes, flycatchers, forest birds). The arthropod model also integrated data from different sampling methods, in this case malaise traps, sweep‐nets, and visual surveys, assuming that each method sampled varying proportions of the true underlying population size (Jarrett et al., 2023; Miller et al., 2019). The model incorporated different capture rates for each method and taxon to allow for varying detection. The estimated population size from this model was at the scale of one tree; we then extrapolated to 1 km^2^ (to match bird spatial scale) by assuming one tree occupies 9 m^2^ (Appendix S1: Section S5).

We calculated the mean and standard deviation of the MCMC chains for each model state (bird or arthropod population size at farm *f =* 1 … 28 and timepoint *t =* 1 … 19) and converted these to biomass by standardizing by body mass of taxa (Appendix S1: Section S5). We provided both the mean and standard deviation as data to the process model, thus explicitly incorporating in the process model the uncertainty in biomass estimates from the observation models.

#### Specifying the food web model for cocoa agroforestry

We modeled community dynamics of birds and arthropods in cocoa as described in Equations ((1), (2), (3), (4), (5))–((1), (2), (3), (4), (5)). The number of interacting taxa (*I*) was 18 (Figure 2).

We modeled the log of the intrinsic growth rate of each taxon *i* as a function of two independent variables: shade cover at each farm *f* and the season during which timepoint *t* fell (Equation 6; categorical variable: ${\text{season}}_{t}=0$ for dry season and ${\text{season}}_{t}=1$ for wet season). This assumes that a species caught in two different farms with equal shade cover and during the same season would have equal intrinsic growth rates. We constrained $b_{\mathrm{ift}}$ to be positive for arthropods to replicate the common food web parametrization for primary producers, in the absence of lower trophic levels (Table 1; Pimm, 1982) and negative for birds (Table 1). Parameters describing the effects of covariates on the log of intrinsic growth rates were given normally distributed priors centered around 0 (Table 1), allowing for the effect of shade and season to be positive or negative.

(6) $$b_{\mathrm{ift}}=\nu_{i0}+\nu_{i1}{\text{canopy}}_{f}+\nu_{i2}{\text{season}}_{t}$$

**TABLE 1:** Description of parameters estimated by field data model, including model priors and the justification for prior distribution.

Parameter	Description	Prior	Justification of prior
ν_prey0_	Intercept of linear predictor of log of intrinsic growth rate for prey groups	Γ(25,16.6)	Doubling time of arthropod population 10–30 days^a^
ν_predator0_	Intercept of linear predictor of log of intrinsic growth rate for predator groups	ν_predator0_ = _−_ν_predator0_pos_ ν_predator0_pos_ ~ Γ(17.2,4.2)	Half‐time for bird population ranging 2–10 days^b^
ν_*i*1_	Effect of canopy on log of intrinsic growth rate	N(0,0.5)	
ν_*i*2_	Effect of season on log of intrinsic growth rate	N(0,0.5)	
*a*_predator,prey_	Interaction coefficient for prey on predator	Γ(1,20)	Bird energy requirements ~4.2 kJ/g, insect energy content ~6.75 kJ/g wet matter, insect water content 0.7, bird energy efficiency 0.75, approximate number of insect groups consumed = 7, a_prey,predator_ = consumption/N*_prey_, a_predator,prey_ = a_prey,predator_ × energy efficiency^c^
*a*_prey,predator_	Interaction coefficient for predator on prey	−*a*_predator,prey_/ε	
*a*_*i,i*_	Density dependence coefficient	Γ(1,20)	
ε	Energy efficiency of predators	Βeta(29,13)	Bird energy efficiency 0.75^d^

^a^ ν_prey0_ = log(2)/(doubling time [days]/30).

^b^ ν_predator0_ = log(0.5)/(half‐time [days]/30).

^c^ Gibb (1957).

^d^ Nyffeler et al. (2018).

For the inter‐specific interaction parameters, we assumed no direct competition, given differences in foraging niches among species (Appendix S1: Section S3). While we included no direct interactions within trophic levels, this does not exclude indirect effects, which are captured by the model in the inverse of the interaction matrix (Equation 4).

Of the 72 potential predator–prey interaction parameters (12 prey × 6 predators), we set 25 to 0 based on evidence from diet metabarcoding data from birds that indicated minimal trophic links between certain bird genera and arthropod orders (Appendix S1: Section S4). Importantly, for all other interactions, we did not consider information from diet metabarcoding because quantifying interaction strength based on such diet data is complicated due to primer bias and other nuances of metabarcoding methods (Deagle et al., 2019). Instead, we modeled the remaining consumption parameters $a_{\text{predator},\text{prey}}$ using Gamma priors, assuming that the effect of prey on predator growth rate was positive (Table 1). We restricted $a_{\text{prey},\text{predator}}$ to negative values and scaled with an (estimated) energy efficiency coefficient (Table 1), to represent the negative effect of predators on the growth rate of prey, and the process of energy exchange from prey to predator.

Modeling community dynamics in discrete time can be complicated by the lack of instantaneous feedback loops and instabilities caused by the implicit time lags in the discrete time formulation (Caswell & Neubert, 2006). This can cause explosive model behavior and, consequently, difficulty with fitting the model to data. While our data were collected approximately every six months (January–February and August–September), we fit the model to linearly interpolated monthly time steps to avoid such difficulties. This resulted in 19 total time steps, spanning January 2019 to August 2020.

We ran the process model with three chains for 50,000 MCMC iterations (plus 10,000 burn‐in). We fit all models using Bayesian inference with the JAGS 4.3.0 software (Plummer, 2017) executed using the runjags package (Denwood, 2016) in the R statistical computing environment (R Core Team, 2023).

#### Quantifying the effects of management on long‐term community composition

To understand long‐term effects of shade cover on community composition of birds and arthropods, we used the posteriors of parameters for growth rate and species interactions. We drew 1000 MCMC equally spaced trials from our total set of MCMC iterations approximating the joint posterior and used these draws to simulate community trajectories under different shade cover values over a 20‐year period, following Equations ((1), (2), (3))–((1), (2), (3)) and (6). To initialize those simulations, we used the mean of the posterior distribution for the density of each taxon (converted to biomass) from the observation models.

#### Predicting changes in community composition under pesticide application scenarios

We wished to investigate the long‐term community‐wide effects of artificially suppressing arthropod populations, as might occur through the application of pesticides or other control measures. Using the parameters estimated from fitting the community model to data, we simulated trajectories over a 20‐year period, as above, but under different scenarios of arthropod population suppression: we simulated nine scenarios, ranging from 10% to 90% impacts on arthropod intrinsic growth rates. This broad range of potential pesticide effects was chosen because pesticide‐induced mortality can vary according to the pesticide product and the arthropod taxon (e.g., from empirical studies, 0.07–0.98 survival rate after 1‐day chemical application, Janssen & van Rijn, 2021). We compared the arthropod suppression scenarios with a scenario with no perturbation; therefore, we assumed that chemical application in our field sites was negligible based on information provided by the farmers. We assumed that chemical application, and therefore growth rate reduction, occurred once per year, and the other time steps in the year were parametrized using the original parameter posteriors with no reduction. Additionally, we assumed that all arthropods were affected equally by pesticide application; this is likely a simplification of what occurs in the field but nonetheless provides useful insight into the food‐web‐wide effects of arthropod declines.

## RESULTS

### Shade cover affects equilibrium food web composition: more forest birds and fewer brown capsids under dense shade

**FIGURE 3:**
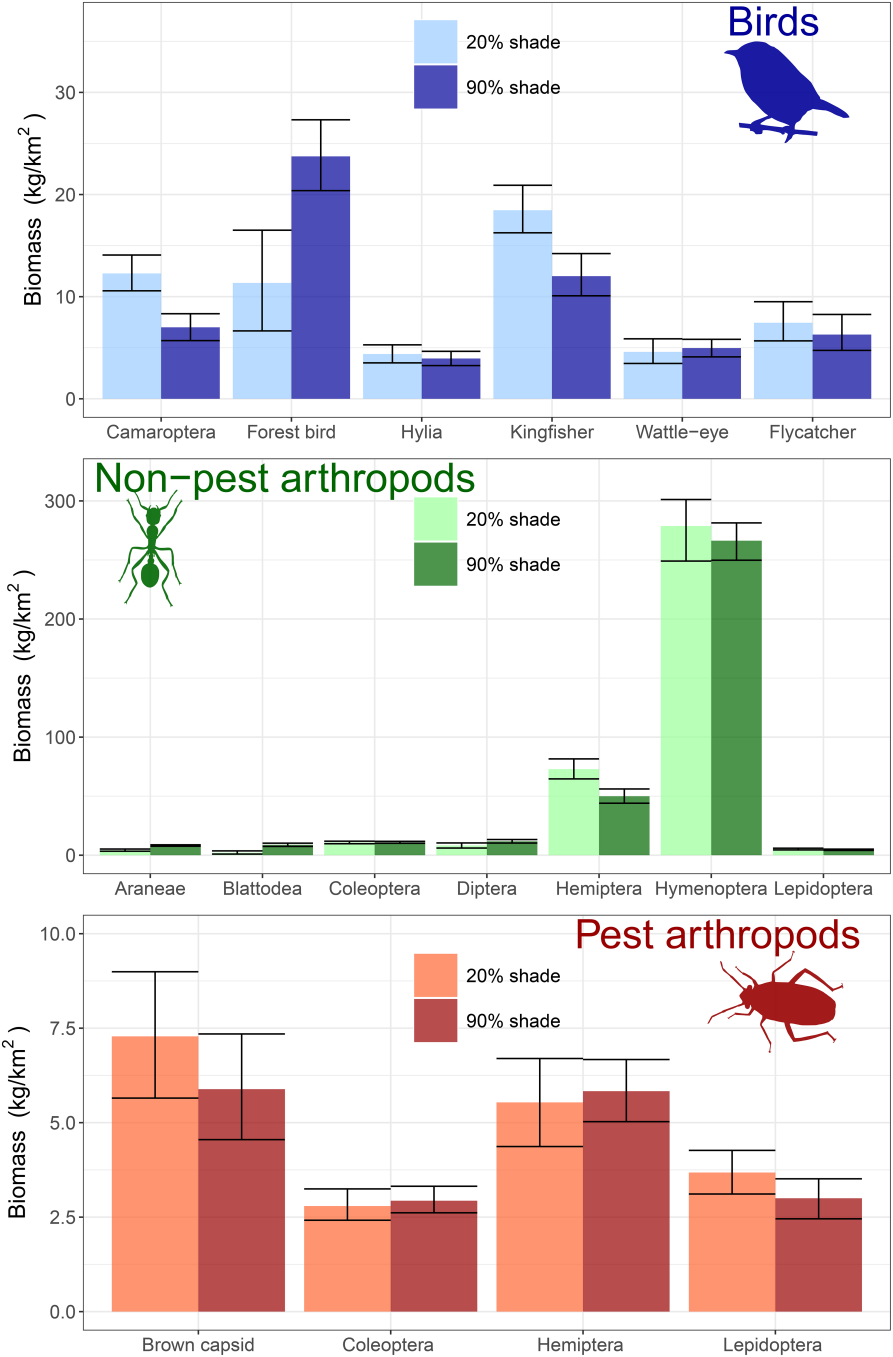
Biomass of taxa after 20 years under low (20%) and high (90%) shade cover. Biomass was calculated at each timepoint using Equations ((1), (2), (3))–((1), (2), (3)) and (6), with parameters estimated from fitting the model to field data. The final timepoint, presented here, represents biomass during the dry season. Bars represent mean biomass values, and error bars capture 95th quantiles. Illustrations were obtained from phylopic.org under license CC0 1.0 (public domain).

Over a 20‐year period, communities settled at different compositions according to shade cover (Figure 3): at low shade cover, the bird community was dominated by kingfishers, followed by camaroptera and forest birds. At high shade cover, bird communities were dominated by forest birds, with at least two times more biomass in this group than any other bird group. Under all shade scenarios, wattle‐eyes, hylia, and flycatchers occurred at low biomass, though flycatchers and hylia showed a slight decrease in biomass with increasing shade and wattle‐eyes showed the opposite trend.

Non‐pest arthropod communities under low shade had higher biomass of Hemiptera, Hymenoptera, and Lepidoptera, while all the other groups showed higher biomass under high shade cover. Araneae and Blattodea showed the steepest increase in biomass with shade cover. For the pest community, under low shade, biomass of capsids and Lepidoptera pests was higher, and biomass of hemipteran and coleopteran pests was marginally lower.

After a 20‐year period, biomass of brown capsids was on average 7.5 kg/km^2^ (0.08 kg/ha) in low shade farms and 6 kg/km^2^ (0.06 kg/ha) in high shade farms (Figure 3). By contrast, forest bird biomass in low shade farms was on average 12 kg/km^2^ (0.12 kg/ha), while in high shade, it was 24 kg/km^2^ (0.24 kg/ha; Figure 3).

### Changes in food web composition are mediated through interactions between taxa and shade management

**FIGURE 4:**
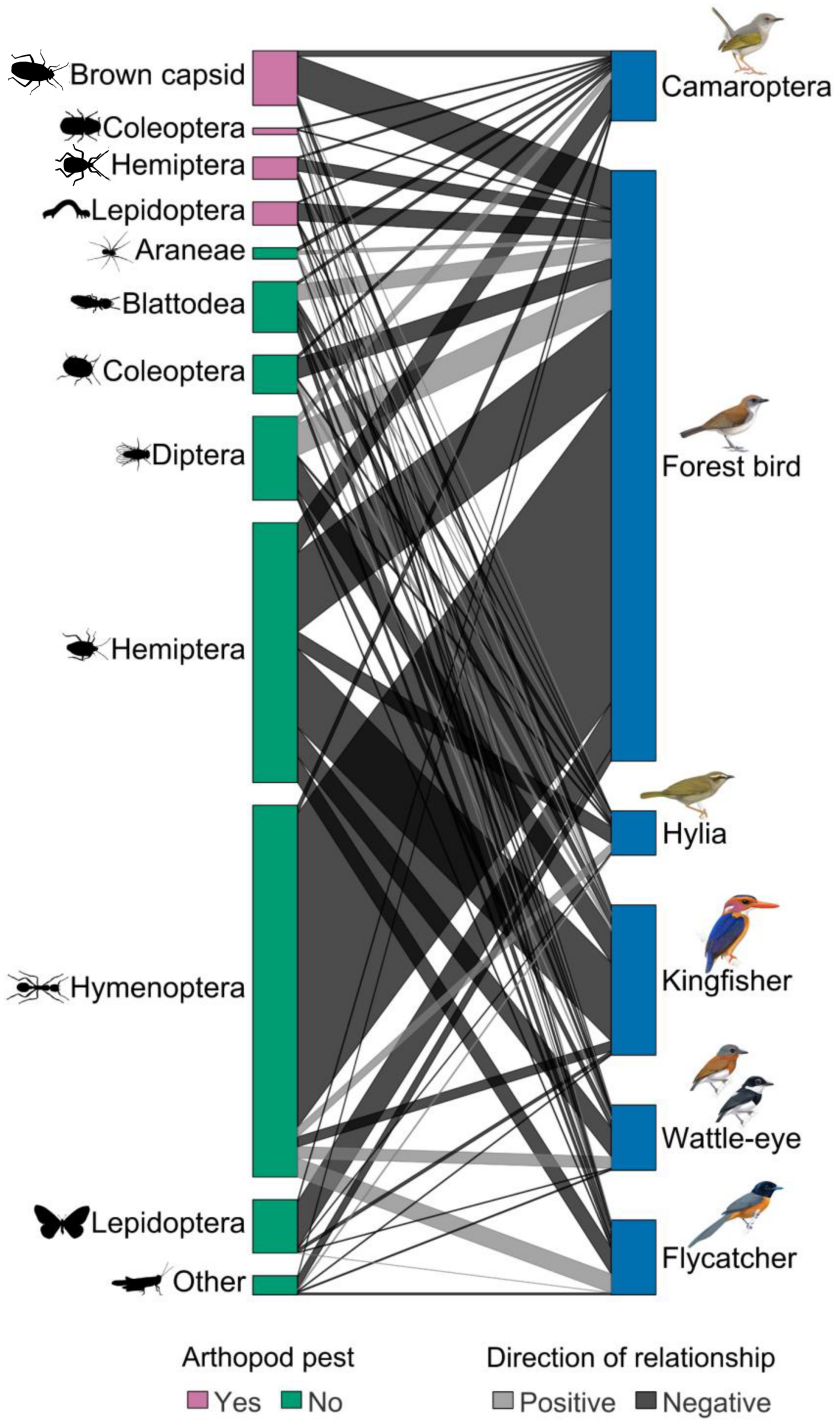
Interactions between bird and arthropod groups in the food web. Represented is the net effect of bird groups on arthropod groups, that is, the change in equilibrium biomass of one taxon caused by the addition of biomass of another taxon, estimated by inverting the interaction matrix (−**A** ^−1^). The thickness of the gray bands and consequently the total height of the boxes representing each species are proportional to the strength of the interactions. Bird illustrations by Faansie Peacock and arthropod illustrations as in Figure 2.

The food web composition under different shade cover values was a result of both the intrinsic response of taxa to shade cover (for ν_*i*1_ posterior summary, see Appendix S1: Section S6) and interactions between taxa (Figure 4; Appendix S1: Section S6). Net effects of each taxon on each other were captured in the inverse of the interaction matrix (**A** ^−1^): in general, the net effects of birds on arthropods were negative and varied in strength. One of the strongest effects was between forest birds and Hymenoptera, and forest birds also had strong negative effects on all pest groups, including brown capsids (Figure 4). In some cases, there were positive interactions between birds and arthropods; these usually occurred between pairs where the direct trophic interaction had been set to 0 based on evidence from diet data, for instance, between forest birds and Diptera. In these cases, as there is no predation, an increase in the biomass of the bird taxon arises through indirect interactions (e.g., by minimizing competition for the non‐prey taxon, Figure 4). While direct effects among bird taxa and among arthropod taxa were set to zero in the model, net effects between bird taxa were all negative, while between arthropod taxa, there were some positive effects.

Importantly, in some cases, the difference in biomass between sunny and shade farms was more strongly influenced by interactions than by intrinsic responses to shade. For instance, the effect of shade cover on the growth rate of hylia was negative (Appendix S1: Section S6), but community states showed almost no change in biomass with shade, indicating that interactions with other taxa (e.g., availability of prey, reduced competition) counteracted the direct effect of shade on growth rate.

### Pesticide application is least effective at suppressing brown capsids in shady farms and results in forest bird extinction

**FIGURE 5:**
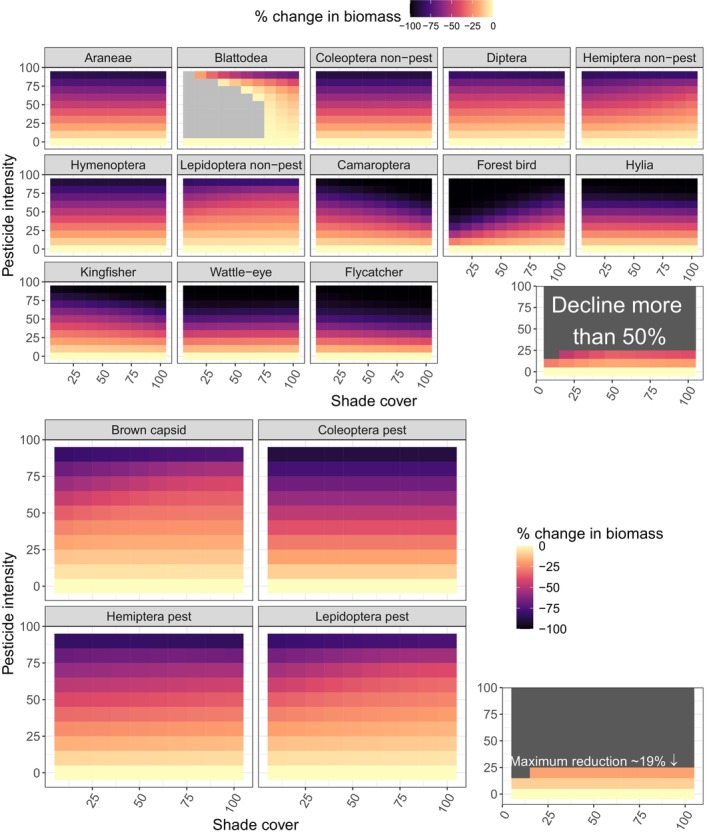
Reduction in biomass (in percentage) in birds and non‐pest arthropods (above) and pests (below) under a range of pesticide intensity and shade cover scenarios for each taxon. Lighter colors indicate less change and darker ones more change. In one case, there is positive change, which is indicated in light gray. The small inlaid panel above summarizes the scenarios where the maximum decline among non‐pest taxa is less than 50%. The inlaid panel below shows, for those same scenarios, the maximum reduction achieved in pest populations.

The long‐term effect of simulated pesticide application on biomass of taxa varied according to shade cover (Figure 5). For brown capsids in low shade farms, a 60% pesticide intensity resulted in a decrease in biomass of −46% (equivalent to 3.3 kg/km^2^), while the equivalent application in high shade farms resulted in a decrease of −30% (2 kg/km^2^). For forest birds, the negative effect of pesticide application was exacerbated in low shade: in sunny farms forest birds became extinct at >40% pesticide intensity, while in shady farms they persisted up to 80% pesticide intensity. Other groups showed varying responses: for instance, camaroptera showed a steeper decrease in biomass with pesticide intensity under shady conditions, while other groups such as Araneae and hylia showed consistent declines in biomass with pesticide intensity independent of shade cover. Interestingly, Blattodea was the only taxon that increased in biomass with pesticide application. Overall, to achieve a decline of less than 50% in non‐pest taxa, pesticide application can only reach 10% in the sunniest farms and 20% in shady farms (Figure 5); this level of application achieves a maximal reduction in pests of 19% (Figure 5).

## DISCUSSION

We investigated the long‐term community‐wide effects of management in agricultural habitats, using African cocoa agroforestry as a model system. We used a community modeling approach, which incorporated information on species' interactions and responses to shade cover. Our results indicate important changes in community composition with management, revealing that low‐intensity farming favors forest bird species and potential pollinators, with no increase in pest biomass.

### Effects of management on long‐term community composition

Equilibrium community composition in sunny farms supports higher biomass of generalist bird taxa, such as *Ispidina* kingfishers and camaropteras, and lower biomass of forest birds. These findings are consistent with previous studies from the same study system and other agroforestry systems around the world, indicating that cocoa farms, including intensively managed ones, can support relatively high diversity of birds, but lose specialized insectivores (Bennett et al., 2021; Faria et al., 2006; Jarrett et al., 2021; Waltert et al., 2005). However, here we go beyond commonly used correlational approaches and predict states of communities in agroforestry from a mechanistic community model that accounts for the influence of species interactions as well as the direct influence of shade cover. This reveals that the response of taxa to shade cover is driven both by an intrinsic response to shade (captured by the $\nu_{i1}$ parameter) and the effect of other taxa in the community. For instance, hylia showed a negative effect of shade on growth rate, yet the population settled at similar biomass in shady farms compared to sunny ones, indicating a response to other taxa in the community. By contrast, forest birds show an intrinsic positive response to shade cover likely reflecting habitat and microclimate requirements for breeding or foraging (Jirinec et al., 2022; Powell et al., 2015). Our findings suggest that widespread intensification and expansion of cocoa agriculture, as seen, for instance, in much of Côte d'Ivoire, will likely result in a landscape dominated by generalist bird taxa and devoid of forest birds and other sensitive insectivores (Kupsch et al., 2019). Even within potential low‐intensity farms or natural habitats, bird communities in such landscapes will change due to the lack of source populations (Jarrett et al., 2021; Sanderson et al., 2022).

The non‐pest arthropod community in both sunny and shady farms was dominated by Hymenoptera, with slightly higher biomass in sunny farms. Diptera occurred at higher biomass in shady farms, as did Araneae and Blattodea, with the latter two showing the most extreme decline down to close to zero biomass in sunny farms. In general, therefore, shady farms supported higher biomass of potential pollinators (Diptera), natural enemies (Araneae), and other ecosystem service providers as found by Ambele et al. (2023). Lepidoptera and Hemiptera decreased in biomass with increasing shade, mostly driven by an intrinsic effect of shade cover on growth rate, perhaps reflecting microhabitat requirements for breeding or development. Our results suggest that widespread intensification of cocoa agroforestry into monocultures results in broad changes in arthropod community composition, driven by a combination of intrinsic responses to shade cover and inter‐specific interactions. Intensified farms support arthropod communities with fewer potential pollinators and natural enemies and near extinction of certain taxa; such changes reflect the global trend of insect declines caused by agricultural intensification (Lister & Garcia, 2018; Outhwaite et al., 2022). Further, there appears to be an additional interactive effect between climate warming and agricultural intensification so that in intensified landscapes, insect declines due to climate warming are exacerbated (Outhwaite et al., 2022). Together, these findings indicate the high risks associated with agricultural intensification. Especially in small‐holder agriculture, where farmers have limited resources and depend heavily on services provided by wild fauna, a decline in important arthropod groups could lead to unsustainable losses.

In the pest community, brown capsids and lepidopteran pests persisted at higher biomasses in sunny farms, while hemipteran and coleopteran pests showed the opposite trend. However, these differences were relatively small with overlapping quantiles potentially due to limited data on pest populations (brown capsids are notoriously hard to detect in the field). Our results indicated that both brown capsid and lepidopteran pests likely had lower growth rates in shady farms; additionally, both groups were consumed by several bird taxa, including forest birds and wattle‐eyes, which were more abundant in shady farms. Indeed, our results suggest that forest birds may have a stronger impact on pest populations than other bird taxa, thus increasing the value of maintaining shade tree cover and forest patches on the landscape (Jarrett et al., 2021). Biological control by native predators in our study sites likely constitutes an important service for farmers with limited resources for agricultural inputs (Ferreira, Jarrett, et al., 2023). Previous studies have found a higher density of brown capsids in sunny farms (Babin et al., 2010; Bagny Beilhe et al., 2018), and such trends could explain long‐term yield declines seen in intensified African cocoa farms (Ahenkorah et al., 1987).

### Changes in community composition under pesticide application scenarios

Under simulated pesticide application scenarios, assumed to reduce arthropod growth rates, we found that shade had an influence on the outcomes of these interventions. Medium levels of pesticide application in sunny farms resulted in the extinction of forest birds likely due to a reduction in prey populations; indeed, forest birds showed the highest susceptibility to chemical use among all taxa, becoming extinct even in shady farms at higher intensity applications. Arthropods themselves rarely became extinct except at the highest level of chemical application but also responded varyingly according to shade. Importantly, applying pesticides in shady farms did little to suppress brown capsid populations likely due to a die‐off of predators permitting population persistence. Overall, this investigation suggested that pesticide use, especially in shady farms, is largely ineffective because it leads to the extinction of predators and consequent higher survival of pest arthropods. These findings suggest limitations of laboratory testing of pesticides; while in laboratory conditions pest populations may be easily eradicated by chemicals, outcomes may change in the presence of other taxa such as predators. Our results support previous modeling work indicating that the effectiveness of pesticide application is strongly mediated through interactions with other taxa (Janssen & van Rijn, 2021). Further experimental work in a field setting, such as manipulation of pesticide applications, is necessary to fully understand the community‐level implications of chemical usage in farms.

The findings from our pesticide simulations match an experimental study conducted in the same study sites, showing that insectivorous birds and bats greatly enhance agricultural yields but only under high shade conditions (Ferreira, Jarrett, et al., 2023). These analyses both suggest that natural pest control by birds and bats may be highly effective in shady farms, whereas productivity will decline (Ferreira, Jarrett, et al., 2023) and pest communities persist in their absence. Overall, recent research globally clearly supports the importance of low‐intensity agriculture and agricultural landscapes in the maintenance of ecosystem services (Bommarco et al., 2013; da Silva et al., 2024; Dainese et al., 2019).

### General considerations on the modeling framework

Together, our results provide robust evidence that intensification of agroforestry results in declines in ecosystem service‐providing arthropods and the high risk of forest bird extinction, which could lead to important losses in pest control services. We also provide a novel framework for ecosystem‐level management, an often‐aspired to but rarely achieved practice (Brussard et al., 1998; Duru et al., 2015). Our intention was to test a relatively simplified version of our food web model to minimize the number of parameters required to be estimated. This simplified scenario has its limitations while also providing opportunities for extensions and further development. Some of the most important limitations of our model include the simplified interaction structure (e.g., no interactions within trophic levels), the lack of a direct link to crop productivity, and the taxonomic clustering.

Extensions to this model could therefore include more types of species interactions (e.g., capturing important ecosystem services such as pollination or predation between arthropod groups), as well as additional predators (e.g., insectivorous bats). An important extension to the model would be to add crops as a node in the food web; this would allow direct estimation of changes in crop biomass as a function of management and food web composition. Additional covariates that account for landscape‐level processes should also be considered, to capture differences in population dynamics according to landscape composition (e.g., proximity to source populations). Structural features such as non‐linear functional responses, prey switching, and Allee effects could be explored via non‐linear extensions of our GLMs (Asseburg et al., 2006; Lindmark et al., 2019). Such non‐linear extensions could result in more equilibria, though this remains to be tested. The taxonomic clustering (mostly genus level for birds, mostly order level for arthropods) may have led to increased uncertainty in the estimation of certain parameters. For instance, the effect of shade cover within arthropod orders may be highly variable and therefore hard to represent with one single parameter. More exploration is needed to investigate how data sufficiency compares with the level of taxonomic aggregation. The less data there are, the more aggregated a model may need to be, and the more noise the results will contain.

Here, we presented a novel method for investigating population dynamics of complex communities. Applying our method to data from wildlife communities in agroforestry revealed that communities in low‐intensity farms are important both for biodiversity conservation and for productivity. This win–win in low‐intensity agroecosystems sheds light on the risks of pursuing intensified agriculture in an era of biodiversity crisis.

## AUTHOR CONTRIBUTIONS

Crinan Jarrett, Luke L. Powell, Andreanna J. Welch, Daniel T. Haydon, and Jason Matthiopoulos conceived the study; Crinan Jarrett led the writing of the manuscript; Daniel T. Haydon and Jason Matthiopoulos supported the modeling work; Crinan Jarrett, Luke L. Powell, Tabe Tiku Regine Claire, Cyril Kowo, and Diogo F. Ferreira collected the data; and Alma L. S. Quiñones and Andreanna J. Welch conducted and designed the lab methods with contributions also from Crinan Jarrett and Luke L. Powell.

## CONFLICT OF INTEREST STATEMENT

The authors declare no conflicts of interest.

## Supporting information


Appendix S1.

## Data Availability

Bird survey data and model code used for bird observation models (Jarrett, 2022) are available in Figshare at https://doi.org/10.6084/m9.figshare.15825111.v1. Arthropod survey data and model code used for arthropod observation models (Jarrett, 2023) are available in Figshare at https://doi.org/10.6084/m9.figshare.21968318.v1. Additional code and data (Jarrett, 2024) are available in Figshare at https://doi.org/10.6084/m9.figshare.24866001.v2.
